# Differential Diagnosis of Fungal Pneumonias vs. Tuberculosis in AIDS Patients by Using Two New Molecular Methods

**DOI:** 10.3390/jof7050336

**Published:** 2021-04-27

**Authors:** Leticia Bernal-Martínez, Laura Herrera, Clara Valero, Paula de la Cruz, Larisa Ghimpu, Ana C. Mesa-Arango, Gabriela Santoni, Lidia Goterris, Rosario Millán, María José Buitrago

**Affiliations:** 1 Centro Nacional de Microbiología, Mycology Reference Laboratory, Instituto de Salud Carlos III, Majadahonda, 28220 Madrid, Spain; leticiabm@isciii.es (L.B.-M.); ci.valerofernandez@gmail.com (C.V.); pauladelacruzr2@gmail.com (P.d.l.C.); larisa.ghimpu@gmail.com (L.G.); ana.mesa@udea.edu.co (A.C.M.-A.); gasantoni18@gmail.com (G.S.); lidiagoterris@gmail.com (L.G.); 2 Centro Nacional de Microbiología, Mycobacteria Unit, Instituto de Salud Carlos III, 28220 Madrid, Spain; lherrera@isciii.es; 3 Department of Microbiology, Hospital Universitario Puerta de Hierro, 28220 Madrid, Spain; mariarosario.millan@salud.madrid.org

**Keywords:** differential diagnosis, tuberculosis, fungal pneumonia

## Abstract

Opportunistic fungal pneumonias (OFP) are the main cause of death in AIDS patients worldwide. Diagnosis of these infections is often late as tuberculosis (TB) is frequently the first suspicion. In addition, diagnostic tools have limitations and are unavailable in disadvantaged regions. To perform the differential diagnosis of the main fungi causing OFP in AIDS patients (*Histoplasma capsulatum*, *Cryptococcus neoformans*/*C. gattii* and *Pneumocystis jirovecii*) vs. the *Mycobacterium tuberculosis* complex (MTBC), two new assays were developed: (i) a multiplex real-time PCR (MRT-PCR) and (ii) a simple and cost-effective method based on real-time PCR and the analysis of melting curves after amplification (MC-PCR). Both of the techniques were optimized and standardized “in vitro”, showing a suitable reproducibility (CV ranged between 1.84 and 3.81% and 1.41 and 4.83%, respectively), a 100% specificity and detection limits between 20 and 2 fg of genomic DNA per 20 µL of reaction. A validation study was performed by retrospectively using 42 clinical samples from 37 patients with proven fungal infection or TB, and 33 controls. The overall sensitivity for the MRT-PCR assay and the MC-PCR assay was 88% and 90.4%, respectively. Both techniques were fast, sensitive and reproducible, allowing for the detection of these pathogens and the performance of a differential diagnosis.

## 1. Introduction

According to most recent data from the WHO, around 38 million people worldwide live with HIV (http://www.who.int/hiv/data/en/ accessed on 30 March 2021). Opportunistic pneumonias are one of the most important complications in AIDS patients. It has been reported that almost 70% of people infected with HIV suffer lung disease during the course of the infection. Especially when the number of T-CD4 cells decreases, pneumonia causes high morbidity and mortality [1]. Tuberculosis (TB) is one of the diseases most often described in these patients, but opportunistic pneumonias caused by fungi are also a very frequent cause of mortality [2,3,4]. In fact, it has been reported that in one year, fungal infection deaths in AIDS patients represented 47% compared to 24% for TB and the most common causes of fungal pneumonia-associated deaths were cryptococcosis, *Pneumocystis jirovecci* pneumonia (PCP) and disseminated histoplasmosis (in endemic regions) [5].

Opportunistic fungal pneumonias (OFP) such as those caused by *C. neoformans*/*C. gatii*, *P. jirovecii* and *H. capsulatum* are clinically and radiologically similar to each other, and also to TB, which is usually the first clinical suspicion [6,7]. This fact delays the correct diagnosis with a great impact on the outcome of the patient [8,9]. Moreover, these mycoses occur mainly in developing countries where adequate diagnostic tools are not always available [5,10]. It has been reported that around 26% of HIV patients with disseminated histoplasmosis also had mixed infections, TB being the most commonly associated infection [11,12]. Several cohorts of patients with histoplasmosis/TB coinfection and advanced HIV were reviewed by Caceres et al. [13]. Other coinfections involving TB and these fungal pathogens have also been reported [14,15,16]. Therefore, even if TB is detected, mixed infections with fungal pathogens should be ruled out, hence the importance of diagnostic techniques that allow performing a simultaneous detection.

Differential diagnosis of these mycoses with TB is complex as classical methods such as direct visualization and culture exhibit several limitations. Fungal cultures are frequently slow-growing and have low sensitivity, and visualization of fungal structures requires skilled personnel. Serological techniques have variable sensitivity depending on the patient’s immune status and clinical picture [17,18]. Molecular techniques are very promising tools but not always available in resource-limited regions due to the complexity of the assays and the cost of the reagents. To date, no molecular method has been described to be implemented for the differential diagnosis of these mycoses with TB in a single tube. 

For the detection of TB, several methods based on qPCR have been reported targeting different genetic regions: the ITS region of the rDNA, the 16S RNA gene, insertion sequences such as IS6110 and drug-resistant genes such as the *rpoB* gene [19,20,21]. Assays based on the detection of *M. tuberculosis* IS6110 in an open polyvalent PCR platform have been recently reported, such as Abbott RealTime MTB (Abbott, Chicago, IL, USA), and the use of this target has been described as a valuable option for routine molecular diagnosis [22]. Commercial methods are also available, some of them approved by the FDA [23,24].

Recently, the PCR techniques for the detection of *P. jirovecii* have been included in the guidelines of the European Conference on Infections in Leukaemia (ECIL), for PCP diagnosis in non-HIV patients due to their higher sensitivity regarding immunofluorescence-based methods [25]. For the diagnosis of histoplasmosis, molecular techniques are scarce. Some methods based on conventional and real-time PCR have been developed in reference laboratories [26,27,28]. These assays target multicopy regions of rDNA such as the ITS, unicopy genes coding the 100-kDa-like protein and the M antigen. Although results are very promising, no commercial methods are available to date. Finally, for the diagnosis of cryptococcosis, the scenario is similar: few molecular methods have been developed. Recently, the detection of *Cryptococcus* ssp. has been included in the FilmArray Meningitis/Encephalitis panel (BioFire Diagnostics, LLC, Salt Lake City, UT, USA), but the results with these pathogens seem to be weak and have a high proportion of false negatives [29]. Detection of capsular polysaccharide antigen (CPA) is very useful for the diagnosis of cryptococcosis in AIDS patients with cryptococcal meningitis; however, the performance is reduced in patients with only pulmonary involvement [30].

In this work, two new methods for the differential diagnosis of OFP vs. TB were developed with the purpose of achieving a rapid and accurate diagnosis in patients at risk of acquiring these infections. The detection of the *Mycobacterium tuberculosis* complex (MTBC) targeting the IS6110 insertion sequence has been included in a previously developed multiplex real-time PCR assay (MRT-PCR) [31] in order to simultaneously detect four fungal pathogens (*Histoplasma capsulatum*, *P. jirovecii* and *Cryptocccus neoformans*/*C. gattii*) and the species included in the MTBC. Moreover, a second method based on the generation of melting curves after PCR (MC-PCR) was also developed as a cost-effective alternative for resource-limited laboratories. Both methods were validated with a limited number of clinical samples from patients with proven infection (histoplasmosis, PCP, cryptococcosis and TB), as well as control samples, to assess their usefulness. 

## 2. Materials and Methods

### 2.1. Control Strains and Plasmids

DNA from different strains and plasmids was used to standardize both assays (Table 1). All the strains belonged to the Collections of the Mycology Reference Laboratory and the Mycobacteria unit, Spanish National Center of Microbiology. Plasmids were constructed in the Mycology Reference Laboratory. In addition, we used genomic human DNA (Promega, Madrid, Spain) to test specificity. 

### 2.2. Primer and Probe Design

MRT-PCR assay: For the detection of *H. capsulatum* and *C. neoformans/gatii*, primers and molecular beacon probes used in this work were described previously in Gago et al. [31] and targeted the ITS 2 and ITS 1 regions of rDNA, respectively. New primers and molecular beacon probes targeting the *mtLSU* gene for the detection of *P. jirovecii* and the IS1160 region for the detection of the species belonging to the *M. tuberculosis* complex were designed using the Beacon Designer v.7.0 software (Premier Biosoft, Palo Alto, CA, USA) and synthesized by Sigma-Aldrich (Madrid, Spain). An “in silico” study was performed previously to optimize the selection of the most appropriate regions for primer and probe design. 

MC-PCR assay: The same set of primers as the one described above was used. However, we modified the forward primer for the amplification of *H. capsulatum* (ITS region) with the purpose of obtaining a PCR amplicon with a suitable melting temperature that allowed for differentiating all pathogens. The new primers and probes designed in this work are listed in Table 2.

To ensure their specificity, the designed primers and probes were subjected to a BLAST search within the GenBank sequence database (http://blast.ncbi.nlm.nih.gov/Blast.cgi, accessed on 1 February 2017) and the database of the Mycology Reference Laboratory of the Spanish National Center for Microbiology (which contains more than 10,000 distinct sequences). Primers and probes for the amplification of the internal control were previously described [32].

### 2.3. PCR Assay

MRT-PCR assay: The amplification was carried out in a Bio-Rad CFX96 system (Bio-Rad, Madrid, Spain). The fluorophores used to label the molecular beacon probes were: FAM for *H. capsulatum*, HEX for *P.jirovecci*, CY5 for *C. neoformans/gatii*, CY5.5 for the *M. tuberculosis* complex and ROX for the probe detecting the internal control. PCR reactions were performed in 20 µL of final volume containing: 2× SensiMix Probe no ROX (Bioline, Ecogen, Madrid, Spain), 0.5 µM of each primer for each of the four pathogens, 0.25 µM of each internal control primer, 0.4 µM of *P. jirovecii* and *M. tuberculosis* probes, 0.2 µM of *H. capsulatum* and *C. neoformans*/*C. gattii* probes and 0.1 µM internal control probe. MgCl2 was added at a final concentration of 4.5 mM. An amount of 2 fg of the internal control plasmid (pICJF) was also added. As a template, 2 µL of genomic DNA or 4 µL of DNA extracted from the clinical samples was used. The PCR conditions were as follows: an initial step of 95 °C for 10 min, followed by 50 cycles of 95 °C for 25 s, 52 °C for 30 s and 72 °C for 30 s. Each experiment was performed in duplicate and included quantification standards as well as negative controls.

**Figure 1:**
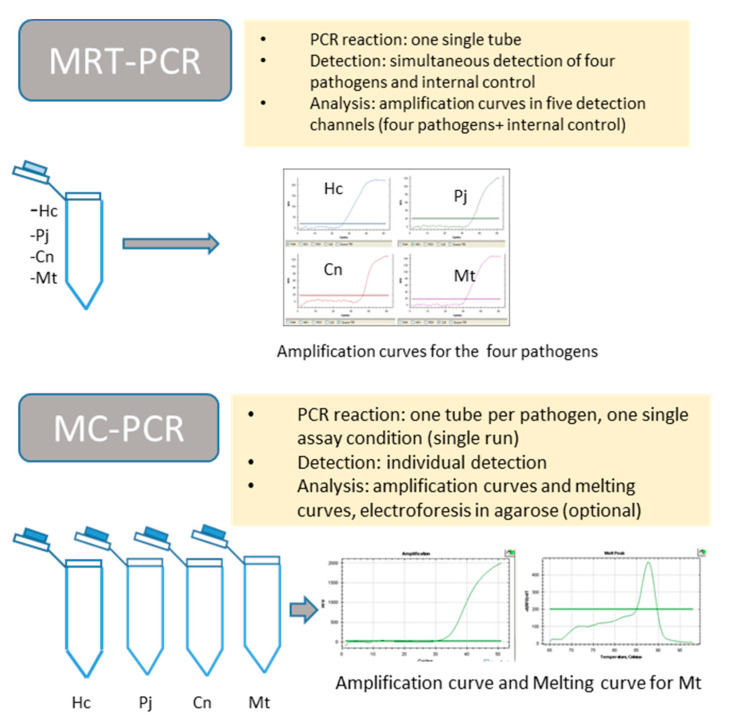
Concept of each assay, pointing out the differences between them. MRT-PCR, multiplex real-time PCR; MC-PCR, melting curve PCR.

MC-PCR assay: The amplification was carried out in a Bio-Rad CFX96 system (Bio-Rad, Madrid, Spain). One individual mix reaction was performed for each pathogen and the internal control. The reactions were performed in 20 µL of final volume containing a pair of primers (0.5 µM), 2× SensiMix Probe no ROX (Bioline, Ecogen, Madrid, Spain) and 1 µl of ResoLight High Resolution Melting Dye (Roche Diagnostics, Madrid, Spain). As a template, 2 µL of genomic DNA or 4 µL of DNA from clinical samples was used. The PCR conditions were as follows: an initial step of 95 °C for 10 min, followed by 50 cycles of 95 °C for 25 s, 59 °C for 30 s and 72 °C for 30 s, and a melt curve was generated by heating from 65 to 98 °C. Fluorescence data were measured every 5 s at 0.5 °C increments. Both assays are summarized in Figure 1. 

### 2.4. Standardization of Both Techniques

Standard curves for *C. neoformans*, *H. capsulatum* and MTBC using both MRT-PCR and MC-PCR methods were constructed based on the results of at least five repetitions with dilutions of genomic DNA ranging from 20 ng to 2 fg in 20 µL of final volume. The strains or plasmids used to construct the standard curves are listed in the Control Strains and Plasmids section. For *P. jirovecii*, a fragment of 124 pb containing the *mtLSU* target region was cloned into a pGEMT-Easy plasmid (Promega, Madrid, Spain) according to the manufacturer’s instructions. A 10-fold serial dilution of this plasmid clone (pSG4), from 20 ng (29.2 log109 copies/20 µL reaction mixture) to 2 fg (29.2 copies/20 µL reaction mixture), was used to construct the standard curve. 

Regression lines were constructed by plotting the logarithm of the initial template concentration vs. the corresponding Cq value. If the coefficient value was >0.980, the standard curve was then used to determine the detection limit and the reproducibility of the assay by calculating the coefficient of variation (CV). In order to evaluate specificity, 2 ng of DNA/20 µL of reaction mixture from other mold, yeast and non-tuberculosis *Mycobacterium* species, as well as human genomic DNA, was included in the PCR assay (Control Strains section).

### 2.5. DNA Extraction

DNA extraction from cultures of *H. capsulatum* and *M. tuberculosis* was performed under biosafety level 3 conditions in compliance with Spanish law (Real Decreto 664/1997). For *H. capsulatum*, the method described by Buitrago et al. [33] was used. For *M. tuberculosis*, a suspension of cells was incubated at 95 °C for twenty minutes and the supernatant was recovered. DNA extraction from *C. neoformans* was performed using a phenol–chloroform method [34]. 

DNA extraction from clinical samples was performed by using the QIAamp DNA minikit (Qiagen, Werfen, Madrid Spain) according to the manufacturer’s instructions without any preprocessing step. Elution was performed in 50 µL of elution buffer. Biopsy samples embedded in paraffin were deparaffinized by lavage with 1.5 mL of xylene (100%) followed by two lavages with 1.2 mL of ethanol (96 to 100%) and an incubation at 37 °C to evaporate the remains of the ethanol. Four µL of DNA extracted from each sample was used for each PCR assay.

In three samples from patients with proven TB, a centrifugation at 10,000 rpm for 10 min was performed, the supernatant was discharged and then the pellet was diluted in 200 µL of supernatant, due to the large volume of saline solution involved. After that, DNA was extracted using the Qiamp DNA minikit (Qiagen) as mentioned above.

### 2.6. Validation with Clinical Samples

The usefulness of both assays was assessed by retrospectively using 75 clinical samples from 70 patients. These samples arrived at our institution to perform diagnostic tests and were stored in a biobank at −20 °C after anonymization (Comité de Ética de la Investigación del Instituto de Salud Carlos III; number CEI PI38_2019-v.2.; date of approval 10 June 2019). The period until their use in this work was variable with a maximum of five years. 

A total of 42 out of these 75 samples belonged to 37 patients with proven infection according to the criteria of EORTC/MSG for histoplasmosis, pneumocystosis and cryptococcosis (35). Patients with TB were confirmed by culture or by direct visualization using Ziehl Neelsen staining. Regarding underlying diseases of patients, 40% were AIDS patients, 8% had another kind of immunosuppression, 5% were immunocompetent and no data for the rest were available (47%). 

Samples from 33 patients with pneumonia caused by other fungal species, viruses or bacteria were used as controls: 11 control samples were used for the MRT-PCR validation and 22 additional control samples were included for the MC-PCR testing. In total, 33 control samples were tested with this MC-PCR method. The reason for using a larger number of samples for MC-PCR was due to the characteristics of the technique (use of an intercalating agent instead of specific probes) that makes it less specific. It was necessary to increase the number of control samples for the validation process with this technique. 

Regarding the kind of samples, due to the lack of respiratory samples for some diseases, other kinds of samples were used for the validation of the technique. A total of 23 bronchoalveolar lavages (BAL), 21 biopsies, 7 sputa, 12 bronchoaspirates (BAS), 3 adenopathies, 2 abscesses, 3 cerebrospinal fluids (CSFs), 2 fine needle aspiration punctions (FNAP), 1 exudate and 1 blood sample were used. Samples from the same patient corresponded to the same date of sampling.

Results for each of the five fluorescence channels were analyzed in the MRT-PCR assay. When MC-PCR assay was tested, melting curves were checked and an agarose gel electrophoresis (2%) was performed to confirm the size of the PCR amplicons.

**Figure 2:**
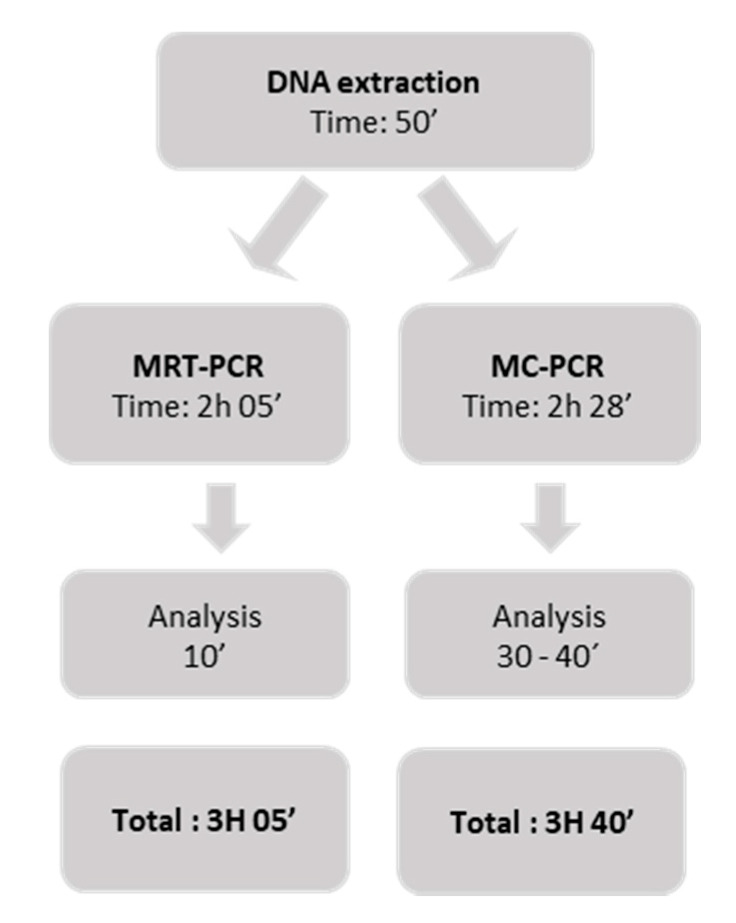
Response time for both methods.

The time required to obtain results with both techniques is presented in Figure 2.

## 3. Results

### 3.1. Standardization of MRT-PCR Assay

**Figure 3:**
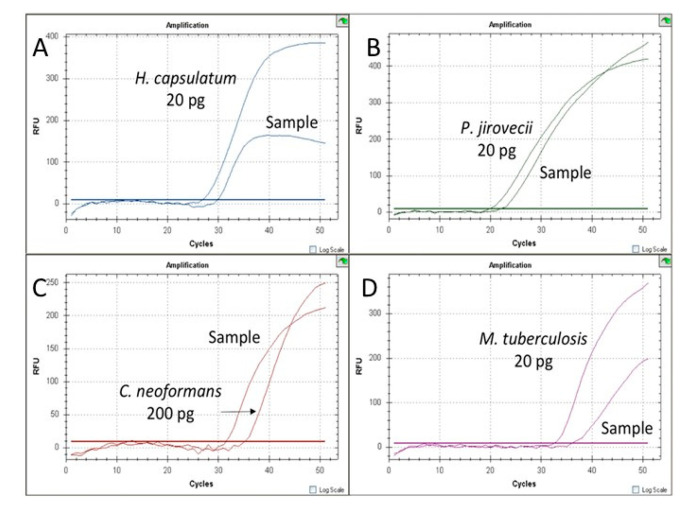
Representative MRT-PCR (multiplex real-time PCR) assay including different positive clinical samples and positive controls. (**A**) *Histoplasma capsulatum* detection, FAM fluorophore; (**B**) *Pneumocystis jirovecci* detection, HEX fluorophore; (**C**) *Cryptococcus neoformans/gatti* detection, Cy5 fluorophore.; (**D**) *Mycobacterium tuberculosis* complex detection, Cy5.5 fluorophore (corresponding to Quasar 705 dye).

The technique specifically and simultaneously detected the four fungal pathogens *H. capsulatum*, *P. jirovecii*, *C. neoformans*/*C. gatii*, the MTBC and an internal control (Figure 3). Each species was detected in its corresponding fluorescence channel (Figure 1). The sizes of the amplicons generated were 124 bp for *P. jirovecii*, 106 bp for *H. capsulatum*, 139 bp for *C. neoformans*/*C. gatii* and 98 bp for the MTBC. The detection limit for *H. capsulatum, C. neoforman/C. gatti* and *M. tuberculosis* was 2 fg of genomic DNA/20 µL of reaction mixture. For *P. jirovecii*, the technique detected up to 29.2 copies/2 µL of reaction mixture. No cross-reactivity to DNA from other fungi or non*-Mycobacterium tuberculosis* species or human DNA (control strains section) was detected. Quantification was linear for the four pathogens included in the assay. The standard curves generated showed a coefficient of determination between 0.94 and 0.99. The reproducibility was suitable as the CV obtained ranged between 1.84 and 3.81%.

### 3.2. Standardization of MC-PCR Assay

**Figure 4:**
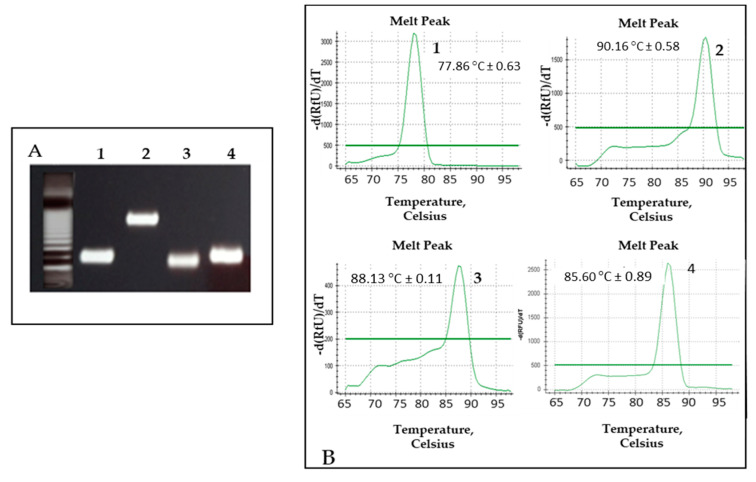
MC-PCR (melting curve PCR) assay results by using DNA from each pathogen. (**A**) Agarose gel electrophoresis with the amplicons generated for each pathogen. Lane: 1. *P. jirovecci*, 124 bp; 2. *H. capsulatum*, 398 bp; 3. *M. tuberculosis* complex, 98 bp; 4. *C. neoformans/gatti*, 139 bp. (**B**) Melting curves generated with the corresponding melting temperature (Tm): 77.86 °C ± 0.63 (*P.jirovecii*); 90.16 °C ± 0.58 (*H. capsulatum*); 88.13 °C ± 0.11 (*M. tuberculosis* complex); 85.60 °C ± 0.89 (*C. neoformans*/*C. gatti*).

The technique specifically amplified the four pathogens and the internal control in five tubes. The amplicons generated had the same size as mentioned above (standardization of MRT-PCR), except for *H. capsulatum* whose amplicon generated by PCR had a size of 398 bp (Figure 4A). Each PCR product generated a different melting curve that was automatically plotted by representing the first negative derivative melting curve (*df/dt*) vs. temperature. The center of the melting peak corresponded to the point of inflection of the melting curve and the melting temperature (Tm) of the PCR product.

The Tms of the PCR products for the different pathogens were: 77.86 °C ± 0.63 (*P. jirovecii*); 85.60 °C ± 0.89 (*C. neoformans*/*C. gatti*); 88.13 °C ± 0.11 (*M. tuberculosis*); 90.16 °C ± 0.58 SD (*H. capsulatum*) (Figure 4B). 

The detection limit for *H. capsulatum*, *C. neoformans*/*C. gattii* and the MTBC was 2 fg of genomic DNA per 20 µL of reaction. For *P. jirovecii*, the technique detected up to 2.92 copies/20 µL of reaction mixture. No cross-reactivity to DNA from other fungi or non-*Mycobacterium tuberculosis* spp. or human DNA (Control Strains section) was detected. The average of the CVs for each pathogen ranged from 1.41 to 4.83%. 

### 3.3. Validation of MRT-PCR Assay with Clinical Samples

A total of 42 clinical samples from 37 patients and 11 control samples were tested. Thirty-seven samples were positive for the pathogen involved in the disease, with a sensitivity of 88.1%, 95% CI, 74.37% to 96.02%. By patients, the assay successfully detected the disease causative agent in 89% (33/37) of them. All samples from patients included as the control population showed negative PCR results, with a specificity of 100%, 95% CI, 71.51% to 100% (Table 3 and Table 4). One histoplasmosis patient and four tuberculosis patients provided a negative result. By samples, the technique was positive in 96% of respiratory samples (23/24), 100% of biopsies (9/9), 67% (2/3) of adenopathies, 50% of FNA (1/2), 50% of abscesses (1/2), one CSF (1/1) and one exudate (1/1).

### 3.4. Validation of MC-PCR Assay with Clinical Samples

A total of 42 clinical samples from 37 patients and 33 controls were tested.

The technique generated the correct melting curve for 38 samples, showing a sensitivity of 90.48%, 95% CI, 77.38% to 97.34% (Table 3). By patients, the assay was positive in 33 out 37 (89.1%). By samples, 92% of respiratory samples (22/24), 100% of biopsy samples (9/9), 67% of adenopathies (2/3) and 50% of abscesses (1/2) were positive. The melting results were confirmed by analyzing the size of the amplicons generated after electrophoresis in agarose gel. Regarding control patients, some unspecific products were amplified with high Cts, but in these cases, the Tm generated served to rule out these products. Gel electrophoresis was also performed for all controls. In five cases, the Tm and the size of the amplicon provided false positive results. Three cases of patients with other respiratory diseases were identified as having PCP (2) and tuberculosis (1), and two cases of patients with invasive aspergillosis were identified as having tuberculosis. All of them were respiratory samples (BAL and BAS). Based on these results, the specificity of the technique was 84.85%, 95% CI, 68.10% to 94.89% (Table 5).

## 4. Discussion

The main fungal pathogens causing opportunistic pneumonia in HIV/AIDS patients are: *H. capsulatum*, *C. neoformans*/*C. gattii* and *P. jirovecii* [3,4]. These infections have a similar clinical picture, mimicking tuberculosis, which is generally the first clinical suspicion, delaying diagnosis and correct treatment [7,9]. In this work, two methods for performing a differential diagnosis of these with TB were developed: (i) an MRT-PCR assay able to detect the aforementioned fungal pathogens and (ii) a simple and cost-effective method based on the analysis of melting curves (MC-PCR). The reason for developing this last method was to offer a simpler alternative to laboratories with low resources. 

The MRT-PCR technique developed was based on another previously published work that showed a good performance for the detection of the four fungal pathogens in clinical samples [31]. In order to optimize the performance of this technique, some modifications were implemented. The target for the detection of *P. jirovecii* (ITS region from ribosomal DNA) was replaced by the *mtLSU* gene. New primers and a probe were designed for that purpose. By using this new target, the detection limit was improved (data not shown). For the detection of the MTBC, a pair of primers and a molecular beacon probe targeting the IS6110 region were designed. This region is specific to the MTBC and is the most abundant multicopy insertion sequence of its genome [35], making it a valuable tool in the diagnosis of TB in biological samples [36]. This region has been used as a target in several commercial and “in-house” PCR assays [36,37]. However, it should be taken into account that it is an insertion region, and its number is variable among strains, affecting the sensitivity of the assay [38].

In the MC-PCR assay, in order to generate an amplicon with a Tm different from the rest, a primer for the amplification of *H. capsulatum* was replaced (Table 1). Therefore, the four pathogens could be clearly distinguished. 

As the results of the “in vitro” standardization, both developed methods had a good sensitivity and reproducibility, and cross-reaction with other fungi or mycobacteria tested was not detected. The techniques were able to simultaneously detect the pathogens in an “in vitro” assay (Supplementary Figure S1). Both techniques were validated by using 75 samples: 42 samples from 37 patients with proven histoplasmosis, cryptococcosis, PCP or TB and 33 control samples. Overall sensitivity in samples from patients with proven infection was 88% for the MRT-PCR assay and 90.4% for the MC-PCR assay. Respiratory samples and biopsies provided an excellent sensitivity in both assays. A total of 100% of biopsies were positives in both assays as were 96% of respiratory samples using MRT-PCR and 92% of respiratory samples using MC-PCR. Regarding specificity, control samples used to validate the MRT-PCR assay were all negative (100% specificity). In the MC-PCR, 5 samples out of 33 controls gave false positive results (84, 85% specificity): two negative samples were positive for *P. jirovecii* and one negative sample and two samples from patients with aspergillosis were positive for the MTBC. In all these false positive results, the analysis of the amplification bands by electrophoresis was useless to rule out an unspecific product. This was expected since the use of an intercalating agent has the disadvantage of decreasing specificity compared to the use of probes; however, this fact should be taken into account as false positives could imply unappropriated treatment. Testing each sample in duplicate could help to confirm the diagnosis. 

False negative results were obtained in four samples from patients with TB by using both techniques; however, samples were not the same in both assays: a sputum and an abscess were negative in both techniques, but one sputum, two adenopathies and one BAL obtained discrepant results (Table 3). The MRT-PCR technique provided a negative result in a respiratory sample from a patient with histoplasmosis. A possible explanation is that the pathogen load was near the detection limit of the techniques in these samples.

Using different kinds of samples for the validation can also affect the overall sensitivity. Respiratory and biopsy samples provided the best results, but other types of samples were not so suitable for the detection of the pathogens. Future studies should be conducted focusing on analyzing sensitivity in different kinds of samples.

To our knowledge, this is the first molecular assay that performed a differential diagnosis of fungal opportunistic pneumonias vs. TB. Although molecular methods for the detection of fungal pathogens have shown their usefulness and have been included in the new guidelines for the diagnosis of invasive fungal diseases [39], there is a shortage of techniques for the detection of species such as *Histoplasma capsulatum* and *C. neoformans*/*C. gattii* and an even greater shortage in the multiplex format including TB detection. The techniques developed are targeted to patients with AIDS from endemic regions with low resources, in which these opportunistic infections have a high incidence and associated mortality. In the context of non-endemic regions, they are also very useful due to the increase in imported mycoses in recent years [40,41,42]. The advantage of these techniques over other techniques, such as LFD for *Cryptococcus* detection or Genexpert for *M. tuberculosis*, is the possibility of detecting the four fungal species and TB simultaneously (coinfections) (FS1) or individually in less than four hours (Figure 2). The main limitation of the MRT-PCR assay is the need for equipment able to detect five different fluorophores and the use of labeled probes, which render this technique more sophisticated and expensive than the MC-PCR assay. On the other hand, the technique could be adapted to the needs of each region and the equipment available just by taking out of the assay those less frequent or interesting pathogens in the epidemiological context. The MC-PCR technique could be easily adapted to laboratories with different capacities, as it requires basic real-time PCR equipment and an intercalant agent, which makes it simple and cost-effective. Although the cost can vary in different regions, it can be estimated that the cost in equipment and reagents would be cut to half compared to MRT-PCR. Moreover, highly qualified personnel are not required as the analysis of results is easy. Finally, the notion of carrying out the detection of each pathogen in a different tube makes it versatile and easily adaptable to the suspicion in each case. However, the use of an intercalant agent decreases specificity. In this work, unspecific amplifications were detected in some negative controls. Analysis of melting curves served to rule out a positive result. Electrophoresis in agarose was also performed to check the product generated. Regardless, in five cases, false positive results were obtained. 

## 5. Conclusions

In conclusion, the techniques developed are fast, sensitive and specific to the differential diagnosis of tuberculosis vs. fungal pneumonias, and they could be adapted to resource-limited laboratories and even be fine-tuned in a point-of-care format by using small and portable PCR systems. Deeper validation work with a higher number of samples is warranted.

## Figures and Tables

**Table 1:** Clinical strains and plasmids used for the standardization of the techniques.

Clinical Strains			
**Name**	**Species**	**Origin**	**Use in This Work**
CNM-CM6019	*Histoplasma capsulatum*	MRL (ISCIII)	Standardization
CNM-CM2721	*Histoplasma capsulatum*	MRL (ISCIII)	Standardization
CNM-CM5659	*Histoplasma capsulatum*	MRL (ISCIII)	Standardization
CNM-CM7057	*Histoplasma capsulatum*	MRL (ISCIII)	Standardization
CNM-CL 2132	*Cryptococcus neoformans*	MRL (ISCIII)	Standardization
CNM-CLH99	*Cryptococcus neoformans*	MRL (ISCIII)	Standardization
CNM-CLWN99	*Cryptococcus neoformans*	MRL (ISCIII)	Standardization
CNM-UM 405	*Mycobacterium tuberculosis* complex	MU (ISCIII)	Standardization
CNM-UM 495	*Mycobacterium tuberculosis* complex	MU (ISCIII)	Standardization
CNM-UM 76	*Mycobacterium tuberculosis* complex	MU (ISCIII)	Standardization
CNM-UM 77	*Mycobacterium tuberculosis* complex	MU (ISCIII)	Standardization
CNM-UM78	*Mycobacterium tuberculosis* complex	MU (ISCIII)	Standardization
CNM-UM 81	*Mycobacterium tuberculosis* complex	MU (ISCIII)	Standardization
CNM-UM 82	*Mycobacterium tuberculosis* complex	MU (ISCIII)	Standardization
CNM-UM 102	*Mycobacterium tuberculosis* complex	MU (ISCIII)	Standardization
CNM-UM 1596	*Mycobacterium tuberculosis* complex	MU (ISCIII)	Standardization
CNM-UM 1689	*Mycobacterium tuberculosis* complex	MU (ISCIII)	Standardization
CNM-CL 5719	*Candida albicans*	MRL (ISCIII)	Specificity
CNM-CL 5683	*Candida parapsilosis*	MRL (ISCIII)	Specificity
CNM-CL5742	*Candida tropicalis*	MRL (ISCIII)	Specificity
CNM-CL3505	*Aspergillus terreus*	MRL (ISCIII)	Specificity
CNM-CMAF237	*Aspergillus fumigatus*	MRL (ISCIII)	Specificity
CNM-CM 3509	*Aspergillus flavus*	MRL (ISCIII)	Specificity
CNM-CM 1627	*Scedosporium prolificans*	MRL (ISCIII)	Specificity
CNM-CM 3035	*Fusarium solani*	MRL (ISCIII)	Specificity
CNM-CM 3020	*Rhizopus oryzae*	MRL (ISCIII)	Specificity
CNM-CM 4244	*Rhizopus microsporus*	MRL (ISCIII)	Specificity
CNM-CM 2908	*Paracoccidioides brasiliensis*	MRL (ISCIII)	Specificity
CNM-CM 2437	*Mucor* spp.	MRL (ISCIII)	Specificity
CNM-UM 406	*Mycobacterium kansasii*	MU (ISCIII)	Specificity
CNM-UM 407	*Mycobacterium avium*	MU (ISCIII)	Specificity
CNM-UM 408	*Mycobacterium abscessus*	MU (ISCIII)	Specificity
CNM-UM 409	*Mycobacterium gordonae*	MU (ISCIII)	Specificity
CNM-UM 410	*Mycobacterium interjectum*	MU (ISCIII)	Specificity
**Plasmids**			
**Name**	**Construction**	**Origin**	**Use in this work**
pICJF *	PGEM-Easy plasmid+jellyfishderivedsequence [32]	MRL (ISCIII)	Standardization
pSG4 *	pGEMT-Easy plasmid+ mtLSU target region	MRL (ISCIII)	Standardization

CNM-CM: Centre National Microbiology, Collection mycelium; CNM-CL: Centre National Microbiology, Collection yeast; CNM-UM: Centre National Microbiology, Unit Mycobacteries; MRL (ISCIII): Mycology Reference Laboratory (Instituto de Salud Carlos III); MU (ISCIII): Mycobacteries Unit (Instituto de Salud Carlos III). * plasmids constructed.

**Table 2:** Sequences of primers and probes designed for *P. jirovecii* and *M. tuberculosis* complex detection.

Primer or Probe	Sequence
OliPJMitoc1 (f)	5′-CAGAAGAATTGTGGTAAGTA-3′
OliPJMitoc2 (r)	5′-CGAGATATTCAGTGCTATAC-3′
PJmitocMB	5′-HEXCGCGATCCGGACTAGGATATAGCTGGTTGATCGCGBHQ1-3′
Oli MTB IS6110 1 (f)	5′-TAGTGCATTGTCATAGG-3′
Oli MTB IS6110 2 (r)	5′-GATCTCAGTACACATCGA-3′
MTBIS6110MB	5′-CY5.5CGCGATCATTACGCCGGGCTGACAGGTAATCAGATCGCG BHQ2-3′

a f, forward; r, reverse; BHQ1, black hole quencher 1; BHQ2, black hole quencher 2.

**Table 3:** MRT-PCR (multiplex real-time PCR) and MC-PCR (melting curves PCR) results for clinical samples from patients with fungal opportunistic pneumonia and TB.

Disease (N° Samples)	Samples	MRT-PCR Positive Results	MC-PCR Positive Results
Histoplasmosis (9)	Respiratory samples (3)	2	3
	Biopsy (6)	6	6
Total	9	8 (88.8%)	9 (100%)
Pneumocystosis (11)	BAL ^a^ (11)	11	11
Total	11	11 (100%)	11 (100%)
Cryptococcosis (5)	BAL ^b^ (1)	1	1
	CSF ^c^ (1)	1	1
	Biopsy (3)	3	3
Total	5	5 (100%)	5 (100%)
Tuberculosis (17)	Respiratory samples (9)	8	8
	Adenopathy (3)	2	2
	Abscess (2)	1	1
	FNAP ^d^ (2)	1	1
	Exudate (1)	1	1
Total	17	13 (76.4%)	13 (76.4%)
TOTAL	42	37 (88.1%)	38 (90.4%)

^a^ bronchoalveolar lavage, ^b^ bronchoaspirate, ^c^ cerebrospinal fluid and ^d^ fine needle aspiration punction.

**Table 4:** MRT-PCR (multiplex real-time PCR) results for control samples.

Disease (Number of Samples)	Samples	MRT-PCR Results (Negatives/Total)
Aspergillosis (1)	BAS ^b^ (1)	1/1
Mucormycosis (2)	Biopsy (2)	2/2
Candidiasis (3)	Blood (1), CSF ^c^ (1), Biopsy (1)	3/3
Paracoccidioidomycosis (1)	Biopsy (1)	1/1
Negative ^a^ (4)	BAL ^d^ (3) BAS (1)	4/4
Total	11	11/11 (100%)

^a^ bronchoalveolar lavage, ^b^ bronchoaspirate, ^c^ cerebrospinal fluid and ^d^ fine needle aspiration punction.

**Table 5:** MC-PCR (melting curves PCR) results for control samples.

Disease (Number of Samples)	Samples	MC-PCR Results (Negatives/Total)
Aspergillosis (8)	BAS ^b^ (5), BAL^c^ (1), Biopsy (2)	6/8
Mucormycosis (6)	Biopsy (5), CSF ^d^ (1)	6/6
Candidiasis (4)	Biopsy (2), CSF ^d^ (1), Blood (1)	4/4
Paracoccidioidomycosis (1)	Biopsy (1)	1/1
Negative ^a^ (14)	BAL ^c^ (9), BAS ^b^ (5)	12/14
Total	33	28/33 (85%)

^a^ samples from patients with other respiratory diseases, ^b^ bronchoaspirate, ^c^ bronchoalveolar lavage and ^d^ cerebrospinal fluid.

## Data Availability

The datasets analyzed during the current study are available from the corresponding author on reasonable request.

## Supplementary Materials

The following are available online at https://www.mdpi.com/article/10.3390/jof7050336/s1, Figure S1: In vitro co-infections assay results.

## References

[1] Gingo M.R., Balasubramani G.K., Kingsley L., Rinaldo C.R., Alden C.B., Detels R., Greenblatt R.M., Hessol N.A., Holman S., Huang L. (2013). The Impact of HAART on the Respiratory Complications of HIV Infection: Longitudinal Trends in the MACS and WIHS Cohorts. PLoS ONE.

[2] Adenis A.A., Valdes A., Cropet C., McCotter O.Z., Derado G., Couppie P., Chiller T., Nacher M. (2018). Burden of HIV-associated histoplasmosis compared with tuberculosis in Latin America: A modelling study. Lancet Infect. Dis..

[3] Armstrong-James D., Meintjes G., Brown G.D. (2014). A neglected epidemic: Fungal infections in HIV/AIDS. Trends Microbiol..

[4] Limper A.H., Adenis A., Le T., Harrison T.S. (2017). Fungal infections in HIV/AIDS. Lancet Infect. Dis..

[5] Denning D.W. (2016). Minimizing fungal disease deaths will allow the UNAIDS target of reducing annual AIDS deaths below 500 000 by 2020 to be realized. Philos. Trans. R. Soc. B Biol. Sci..

[6] Denis B., Lortholary O. (2013). Infections fongiques pulmonaires chez les patients séropositifs pour le VIH. Rev. Mal. Respir..

[7] Rodríguez-Cerdeira C., Arenas R., Morenocoutino G., Vásquez E., Fernandez R.D., Chang P. (2014). Micosis sistémicas en pacientes con virus de la inmunodeficiencia humana/sida. Actas Dermo-Sifiliográficas.

[8] Mabey D., Peeling R.W., Ustianowski A., Perkins M.D. (2004). Diagnostics for the developing world. Nat. Rev. Genet..

[9] Teo A.K.J., Singh S.R., Prem K., Hsu L.Y., Yi S. (2019). Delayed diagnosis and treatment of pulmonary tuberculosis in high-burden countries: A systematic review protocol. BMJ Open.

[10] Nacher M., Adenis A., Aznar C., Blanchet D., Vantilcke V., Demar M., Carme B., Couppié P. (2014). How Many Have Died from Undiagnosed Human Immunodeficiency Virus–Associated Histoplasmosis, A Treatable Disease? Time to Act. Am. J. Trop. Med. Hyg..

[11] Gutierrez M.E., Canton A., Sosa N., Puga E., Talavera L. (2005). Disseminated Histoplasmosis in Patients with AIDS in Panama: A Review of 104 Cases. Clin. Infect. Dis..

[12] Samayoa B., Roy M., Cleveland A.A., Medina N., Lau-Bonilla D., Scheel C.M., Gomez B.L., Chiller T., Arathoon E. (2017). High Mortality and Coinfection in a Prospective Cohort of Human Immuno-deficiency Virus/Acquired Immune Deficiency Syndrome Patients with Histoplasmosis in Guatemala. Am. J. Trop. Med. Hyg..

[13] Caceres D.H., Valdes A. (2019). Histoplasmosis and Tuberculosis Co-Occurrence in People with Advanced HIV. J. Fungi.

[14] Almeida-Silva F., Damasceno L.S., Serna M.J.B., Valero C., Quintella L.P., Almeida-Paes R., Muniz M.D.M., Zancope-Oliveira R.M. (2016). Multiple opportunistic fungal infections in an individual with severe HIV disease: A case report. Revista Iberoamericana de Micología.

[15] Aronis M.L., Dos Santos R.P., Goldani L.Z. (2011). Disseminated Histoplasma capsulatum and Cryptococcus neoformans Co-Infection in Patients with AIDS. Mycopathologia.

[16] Orlovic D., Kularatne R., Ferraz V., Smego R.A. (2001). Dual Pulmonary Infection with Mycobacterium tuberculosis and Pneumocystis carinii in Patients Infected with Human Immunodeficiency Virus. Clin. Infect. Dis..

[17] Hage C.A., Knox K.S., Wheat L.J. (2012). Endemic mycoses: Overlooked causes of community acquired pneumonia. Respir. Med..

[18] Wheat L.J., Azar M.M., Bahr N.C., Spec A., Relich R.F., Hage C. (2016). Histoplasmosis. Infect. Dis. Clin. N. Am..

[19] Babafemi E.O., Cherian B.P., Banting L., Mills G.A., Ngianga K. (2017). Effectiveness of re-al-time polymerase chain reaction assay for the detection of Mycobacterium tuberculosis in patholog-ical samples: A systematic review and meta-analysis. Syst. Rev..

[20] De Vos M., Derendinger B., Dolby T., Simpson J., Van Helden P.D., Rice J.E., Wangh L.J., Theron G., Warren R.M. (2018). Diagnostic Accuracy and Utility of FluoroType MTBDR, a New Molecular Assay for Multidrug-Resistant Tuberculosis. J. Clin. Microbiol..

[21] Tevere V.J., Hewitt P.L., Dare A., Hocknell P., Keen A., Spadoro J.P., Young K.K. (1996). Detection of Mycobacterium tuberculosis by PCR amplification with pan-Mycobacterium primers and hybridization to an M. tuberculosis-specific probe. J. Clin. Microbiol..

[22] Kolia-Diafouka P., Godreuil S., Bourdin A., Carrère-Kremer S., Kremer L., Van De Perre P., Tuaillon E. (2018). Optimized Lysis-Extraction Method Combined With IS6110-Amplification for Detection of Mycobacterium tuberculosis in Paucibacillary Sputum Specimens. Front. Microbiol..

[23] Nurwidya F., Handayani D., Burhan E., Yunus F. (2018). Molecular Diagnosis of Tuberculosis. Chonnam Med. J..

[24] Schito M., Migliori G.B., Fletcher H.A., McNerney R., Centis R., D’Ambrosio L., Bates M., Kibiki G., Kapata N., Corrah T. (2015). Perspectives on Advances in Tuberculosis Diagnostics, Drugs, and Vaccines. Clin. Infect. Dis..

[25] Alanio A., Hauser P.M., Lagrou K., Melchers W.J.G., Helweg-Larsen J., Matos O., Cesaro S., Maschmeyer G., Einsele H., Donnelly J.P. (2016). ECIL guidelines for the diagnosis of Pneumocystis jirovecii pneumonia in patients with haematological malignancies and stem cell transplant recipients. J. Antimicrob. Chemother..

[26] Bialek R., Feucht A., Aepinus C., Just-Nübling G., Robertson V.J., Knobloch J., Hohle R. (2002). Evaluation of Two Nested PCR Assays for Detection of Histoplasma capsulatum DNA in Human Tissue. J. Clin. Microbiol..

[27] Buitrago M.J., Berenguer J., Mellado E., Rodriguez-Tudela J.L., Cuenca-Estrella M. (2006). Detection of imported histoplasmosis in serum of HIV-infected patients using a real-time PCR-based assay. Eur. J. Clin. Microbiol. Infect. Dis..

[28] Martagon-Villamil J., Shrestha N., Sholtis M., Isada C.M., Hall G.S., Bryne T., Lodge B.A., Reller L.B., Procop G.W. (2003). Identification of Histoplasma capsulatum from culture extracts by real-time PCR. J. Clin. Microbiol..

[29] Tansarli G., Chapin K. (2020). Diagnostic test accuracy of the BioFire® FilmArray® meningitis/encephalitis panel: A systematic review and meta-analysis. Clin. Microbiol. Infect..

[30] Baddley J.W., Dismukes W.E. (2013). Cryptococcosis. Essentials of Clinical Mycology.

[31] Gago S., Esteban C., Valero C., Zaragoza O., Puig d.l.B., Buitrago M.J. (2014). A multiplex re-al-time PCR assay for identification of *Pneumocystis jirovecii*, *Histoplasma capsulatum*, and *Crypto-coccus neoformans/Cryptococcus gattii* in sam-ples from AIDS patients with opportunistic pneumonia. J. Clin. Microbiol..

[32] Bernal-Martínez L., Buitrago M.J., Castelli M.V., Rodriguez-Tudela J.L., Cuenca-Estrella M. (2013). Development of a single tube multiplex real-time PCR to detect the most clinically relevant Mucormycetes species. Clin. Microbiol. Infect..

[33] Buitrago M.J., Canteros C.E., De León G.F., Gonzalez A., De Oliveira M.M.-E., Muñoz C.O., Ramirez J.A., Toranzo A.I., Zancope-Oliveira R., Cuenca-Estrella M. (2013). Comparison of PCR protocols for detecting *Histoplasma capsulatum* DNA through a multicenter study. Revista Iberoamericana de Micología.

[34] Tang C.M., Cohen J., Krausz T., Van Noorden S., Holden D.W. (1993). The alkaline protease of *Aspergillus fumigatus* is not a virulence determinant in two murine models of invasive pulmonary aspergillosis. Infect. Immun..

[35] Thierry D., Brisson-Noël A., Vincent-Lévy-Frébault V., Nguyen S., Guesdon J.L., Gicquel B. (1990). Characterization of a *Mycobacterium tuberculosis* insertion sequence, IS6110, and its application in diagnosis. J. Clin. Microbiol..

[36] Brisson-Noël A., Nguyen S., Aznar C., Chureau C., Garrigue G., Pierre C., Bartoli M., Bonete R., Pialoux G., Gicquel B. (1991). Diagnosis of tuberculosis by DNA amplification in clinical practice evaluation. Lancet.

[37] Armand S., Vanhuls P., Delcroix G., Courcol R., Lemaitre N. (2011). Comparison of the Xpert MTB/RIF test with an IS6110-TaqMan real-time PCR assay for direct detection of *Mycobacterium tuberculosis* in respiratory and nonrespiratory specimens. J. Clin. Microbiol..

[38] El Khéchine A., Henry M., Raoult D., Drancourt M. (2009). Detection of Mycobacterium tuberculosis complex organisms in the stools of patients with pulmonary tuberculosis. Microbiology.

[39] Barlow R.E.L., Gascoyne-Binzi D.M., Gillespie S.H., Dickens A., Qamer S., Hawkey P.M. (2001). Comparison of Variable Number Tandem Repeat and IS6110-Restriction Fragment Length Polymorphism Analyses for Discrimination of High- and Low-Copy-Number IS6110 *Mycobacterium tuberculosis* Isolates. J. Clin. Microbiol..

[40] Donnelly J.P., Chen S.C., Kauffman C.A., Steinbach W.J., Baddley J.W., Verweij P.E., Clancy C.J., Wingard J.R., Lockhart S.R., Groll A.H. (2020). Revision and Update of the Consensus Definitions of Invasive Fungal Disease From the European Organization for Research and Treatment of Cancer and the Mycoses Study Group Education and Research Consortium. Clin. Infect. Dis..

[41] Buitrago M.J., Bernal-Martínez B.L., Castelli M.V., Rodríguez-Tudela J.L., Cuenca-Estrella M. (2010). Histoplasmosis and Paracoccidioidomycosis in a Non-Endemic Area: A Review of Cases and Diagnosis. J. Travel Med..

[42] Molina-Morant D., Sánchez-Montalvá A., Salvador F., Sao-Avilés A., Molina I. (2018). Imported endemic mycoses in Spain: Evolution of hospitalized cases, clinical characteristics and correlation with migratory movements, 1997–2014. PLoS Negl. Trop. Dis..

