# Supplementary material for: Differential Diagnosis of Fungal Pneumonias vs. Tuberculosis in AIDS Patients by Using Two New Molecular Methods
Source: J Fungi (Basel). 2021 Apr 27;7(5):336. doi: 10.3390/jof7050336 (PMC8145742; doi:10.3390/jof7050336)
Supplement: Supplementary file 1 [file jof-07-00336-s001.zip › jof-1183950-supplementary.pdf]

Supplementary Figure 1(SF1): *In vitro* co-infections assay results

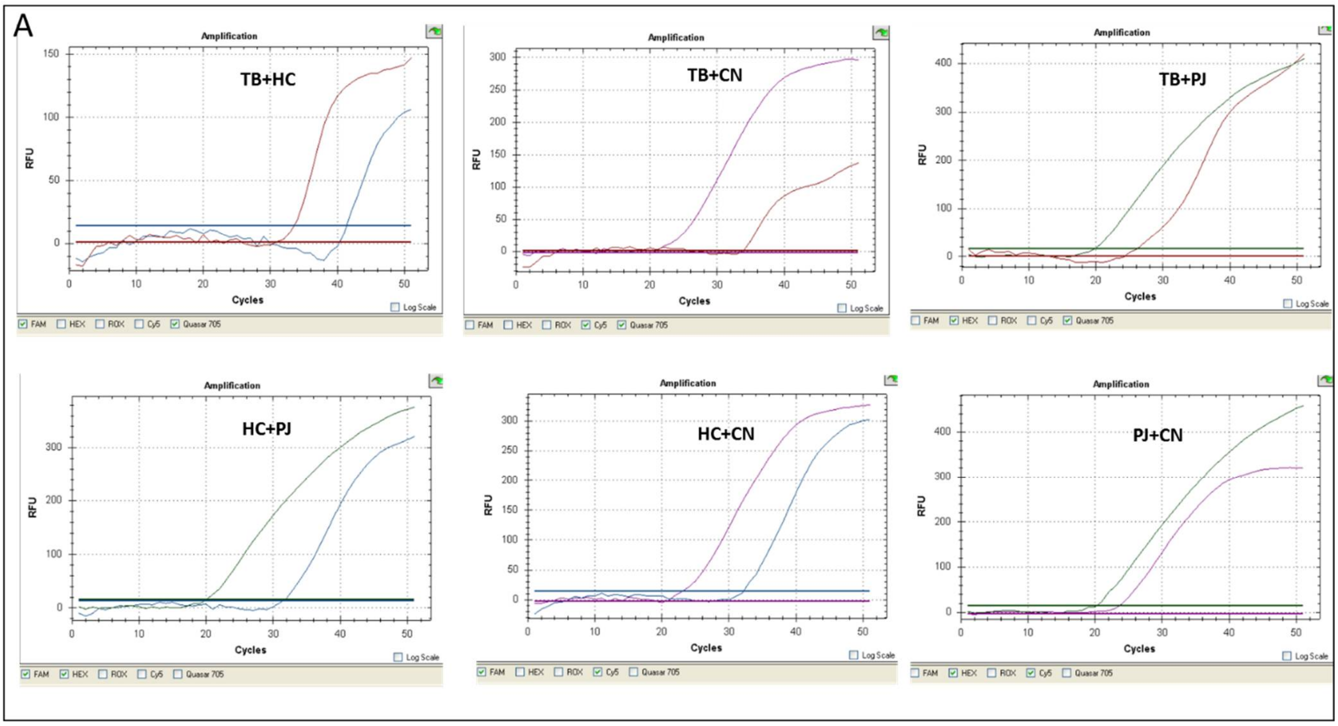

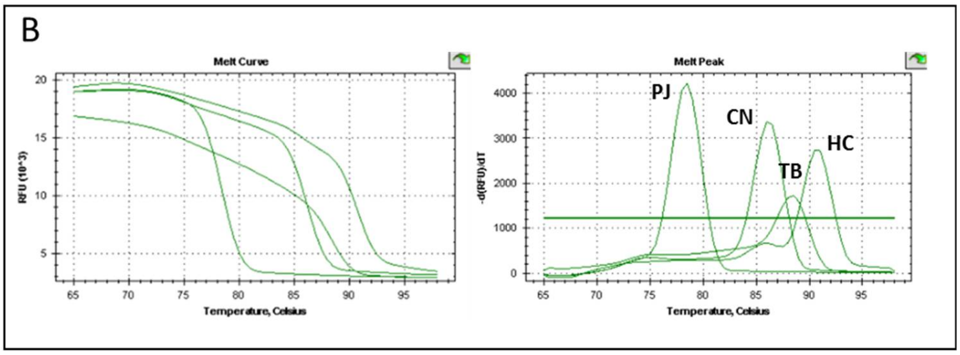

Supplementary Figure 1(SF1): “in vitro” co-infections assay results. A. MRT- PCR. results by using mixtures of 20 fg of different DNAs. Each pathogen amplified in its respective channel. B. MC-PCR results by using mixtures of 20 fg of different DNAs. Each pathogen was tested independently. TB: *Mycobacterium tuberculosis*, HC: *Histoplasma capsulatum*, Pj*: Pneumocystis jirovecci* and CN: *Cryptococcus neoformans*.
